# Impact of anemia on in-stent restenosis after percutaneous coronary intervention

**DOI:** 10.1186/s12872-021-02355-1

**Published:** 2021-11-19

**Authors:** Huilin Hu, Shijun Wang, Guanmin Tang, Changlin Zhai, Liang Shen

**Affiliations:** Department of Cardiology, Affiliated Hospital of Jiaxing University, No.1882 Zhonghuan South Road, Jiaxing, Zhejiang China

**Keywords:** Anemia, In-stent restenosis, Percutaneous coronary intervention

## Abstract

**Background:**

Anemia is a common risk factor for post-percutaneous coronary intervention (PCI) adverse events; however, data on its association with in-stent restenosis (ISR) is limited.

**Methods:**

538 patients who underwent PCI between January 2017 and September 2019 and follow-up angiography 9–12 months after the initial PCI were enrolled in this study. Baseline clinical and procedural characteristics were compared between the ISR and non-ISR groups, and independent predictors of ISR were determined using propensity score matching.

**Results:**

The incidence of anemia was 53.5% in patients with ISR and 19.0% in those without ISR. Univariable logistic regression analyses showed that anemia (OR, 4.283; 95% CI, 1.949–9.410; *P* < 0.001), diabetes mellitus (OR, 2.588; 95% CI, 1.176–5.696; *P* = 0.018), chronic kidney disease (OR, 3.058; 95% CI, 1.289–7.252; *P* = 0.011), multiple stenting (OR, 2.592; 95% CI, 1.205–5.573; *P* = 0.015), bifurcation lesion (OR, 2.669; 95% CI, 1.236–5.763; *P* = 0.012), and calcification (OR, 3.529; 95% CI, 1.131–11.014; *P* = 0.030) were closely associated with ISR. Low-density lipoprotein cholesterol (LDL-c) levels and stent diameter were also significantly linked to ISR, as was anemia (*P* = 0.009) after propensity score matching.

**Conclusion:**

Anemia is closely associated with post-PCI ISR, and patients with lower hemoglobin levels are at a higher risk of ISR.

## Introduction

Percutaneous coronary intervention (PCI) has been an effective and widely used treatment for patients with coronary artery disease (CAD) since 1979 [1]. However, despite ongoing improvements on drug-eluting stents (DES), in-stent restenosis (ISR) remains a major complication of PCI, accounting for approximately 5% of all complications [2, 3]. Multiple factors, including inflammatory response, patient-specific and procedure-related risk factors, are involved in the development of ISR [4].

Anemia is associated with a greater rate of cardiovascular events [5, 6], and its prevalence is comparatively high in patients with CAD. Previous studies have highlighted links between anemia and a higher risk of death, major adverse cardiovascular events (MACE), myocardial infarction (MI), and bleeding post PCI [7–10]. Most recently, an in-hospital hemoglobin drop ≥ 3 g/dL was independently associated with increased 1-year mortality among patients with acute coronary syndrome managed invasively [11].

While studies have demonstrated associations between anemia and fatal adverse events post-PCI, there is yet no data on the relationship between anemia and ISR. In this study, we sought to identify and establish an association between anemia and ISR and determine its independent predictive ability of ISR post-PCI in patients with CAD.

## Materials and methods

### Participants

Our study complied with the principles of the Declaration of Helsinki and was approved by the Ethics Committee of the First Hospital of Jiaxing, Jiaxing, China. Patient inclusion criteria comprised: (1) fulfilling the criteria for PCI; (2) treatment with second-generation DES; (3) agreement to participate in the study. Patient exclusion criteria constituted: (1) dementia or cognitive dysfunction; (2) severe infection or active liver disease; (3) malignancy; (4) severe chronic kidney disease (estimated glomerular filtration rate [eGFR] < 30 ml/min/1.73 m^2^) or severe anemia (hemoglobin levels < 6 g/dL), (5) coagulation disorders, and (6) lost follow-up.

Per our criteria, 538 CAD patients treated with second-generation DES between January 2017 and September 2019 and follow-up angiography 9–12 months after the initial PCI at our hospital were enrolled upon provision of written informed consent.

The patients’ medical history, pharmacotherapy, angiographic characteristics, and blood biochemistry tests for low-density lipoprotein cholesterol (LDL-c), and triglycerides (TG) were collected. Chronic kidney disease (CKD) was characterized by markers of kidney damage or decreased eGFR persisting for > 3 months [12], coronary calcification was assessed angiographically and defined as apparent densities observed within the artery at the site of stenosis [13], and anemia was distinguished by baseline hemoglobin (Hb) levels < 13 g/dL for men and 12 g/dL for women [14].

### Coronary intervention and follow-up assessment

PCI was performed as described previously, and all patient included were treated with second-generation DES. Follow-up angiography was performed 9–12 months after the first PCI. Restenosis was evaluated using the conventional Quantitative Coronary Angiography (QCA) technique. ISR was defined as a luminal narrowing with more than 50% diameter stenosis of a stented coronary segment or within a 5 mm segment proximal or distal to the stent [15].

### Statistical analysis

All statistical analyses were conducted using IBM SPSS 22.0. Data for continuous variables are expressed as the mean ± SD and categorical variables as percentages. Continuous variables were analyzed with *t*-test and categorical variables with *χ*^2^-test. A two-tailed *P* value less than 0.05 was considered statistically significant. Risk factors for ISR were assessed using binary logistic regression analysis, and odds ratio (OR) and 95% confidence interval (CI) were calculated. We employed the propensity score matching model to analyze the impact of covariates on the relationship between anemia and ISR and also scrutinized the receiver operating characteristics (ROC) curve to establish Hb’s accuracy to predict ISR.

## Results

### General characteristics of the patients with and without ISR

Of the 538 selected patients, 28 (5.2%) occured ISR, and 20 underwent repeat revascularization assisted by intravascular ultrasound (IVUS). Baseline characteristics are summarized in Table 1. Overall, the patients’ average age was 62.2 ± 10.2 years (ranging from 29 to 87 years), and 71.4% of the total were male. The rate of diabetes mellitus, CKD, multiple stenting, and bifurcation lesion was significantly higher in anemia patients with ISR (*P* values 0.000, 0.001, 0.035 and 0.000, respectively) than in the non-ISR group. In patients without anemia, ISR occurred more in individuals with lower Hb levels (*P* values 0.011).

**Table 1:** Baseline and procedual characteristics

Variable	Anemia		*P* value	Without anemia		*P* value
	ISR	Non-ISR		ISR	Non-ISR	
Male, n (%)	14 (3.7)	133 (34.6)	0.524	4 (1.0)	233 (60.7)	0.097
Age (years)	67.1 ± 10.9	62.4 ± 11.2	0.094	54.3 ± 10.4	62.1 ± 9.6	0.012
Current smoker, n (%)	7 (3.0)	81 (34.8)	0.457	5 (2.1)	140 (60.1)	0.525
BMI (kg/m^2^)	23.5 ± 3.7	24.6 ± 2.8	0.120	24.3 ± 2.7	24.4 ± 2.8	0.841
Hypertension, n (%)	10 (2.9)	106 (30.5)	0.367	7 (2.0)	224 (64.6)	0.696
Diabetes mellitus, n (%)	11 (9.7)	31 (27.4)	0.000	0 (0.0)	71 (62.9)	0.221
LDL-c (mmol/L)	2.9 ± 1.5	2.5 ± 0.9	0.687	3.6 ± 1.9	2.6 ± 0.9	0.087
TG (mmol/L)	1.3 ± 0.4	1.6 ± 1.0	0.397	1.8 ± 0.8	1.6 ± 0.9	0.576
Hb (g/dL)	104.8 ± 15.7	111.4 ± 14.6	0.072	134.4 ± 10.8	145.8 ± 13.8	0.011
LVEF (%)	56.5 ± 6.8	60.2 ± 5.8	0.013	59.3 ± 9.8	59.9 ± 6.9	0.777
CKD (%)	8 (11.9)	23 (34.3)	0.001	0 (0.0)	36 (53.8)	0.607
ACS (%)	11 (3.7)	85 (28.8)	0.519	4 (1.4)	195 (66.1)	0.352
Multiple stenting, n (%)	9 (5.8)	42 (26.9)	0.035	5 (3.2)	100 (64.1)	0.142
Stent length (mm)	28.8 ± 6.5	29.7 ± 5.1	0.463	28.8 ± 5.6	29.5 ± 5.6	0.695
Stent diameter (mm)	2.9 ± 0.5	3.2 ± 0.5	0.024	3.0 ± 0.4	3.2 ± 0.5	0.262
Multivessel coronary disease (%)	4 (3.9)	23 (22.5)	0.484	2 (2.0)	73 (51.6)	1.000
LMCA, n (%)	1 (8.3)	2 (16.7)	0.275	1 (8.3)	8 (66.7)	0.226
LAD, n (%)	4 (4.9)	13 (15.9)	0.075	3 (3.7)	62 (75.6)	0.396
LCX, n (%)	14 (5.6)	72 (29.0)	0.008	4 (1.6)	158 (63.8)	1.000
RCA, n(%)	4 (1.9)	68 (32.5)	0.129	3 (1.4)	134 (64.2)	0.748
Bifurcation lesion, n (%)	10 (7.2)	30 (21.7)	0.000	3 (2.2)	95 (68.9)	1.000
Calcification, n (%)	2 (7.4)	9 (33.3)	0.307	2 (7.4)	14 (51.9)	0.068
Statins, n (%)	18 (3.5)	152 (29.9)	0.332	8 (1.6)	331 (65.0)	0.053
Aspirin, n (%)	18 (3.4)	154 (29.1)	0.403	10 (1.9)	348 (65.6)	0.811
Clopidogrel, n (%)	9 (3.4)	83 (31.0)	0.880	6 (2.2)	170 (63.4)	0.476
Ticagrelor, n (%)	6 (2.5)	67 (28.3)	0.485	4 (1.7)	160 (67.5)	0.760

*ISR* In-stent restenosis; *BMI* body mass index; *CKD* Chronic kidney disease; *Hb* hemoglobin; *LVEF* Left ventricular ejection fraction; *ACS* Acute coronary syndrome; *LDL-c* Low-density lipoprotein cholesterol; *TG* triglycerides; *LMCA* Left main conronary artery; *LAD* Left anterior descending artery; *LCX* Left circumflex artey; *RCA* Right coronary artery

### Predictors of ISR at follow-up

**Fig. 1:**
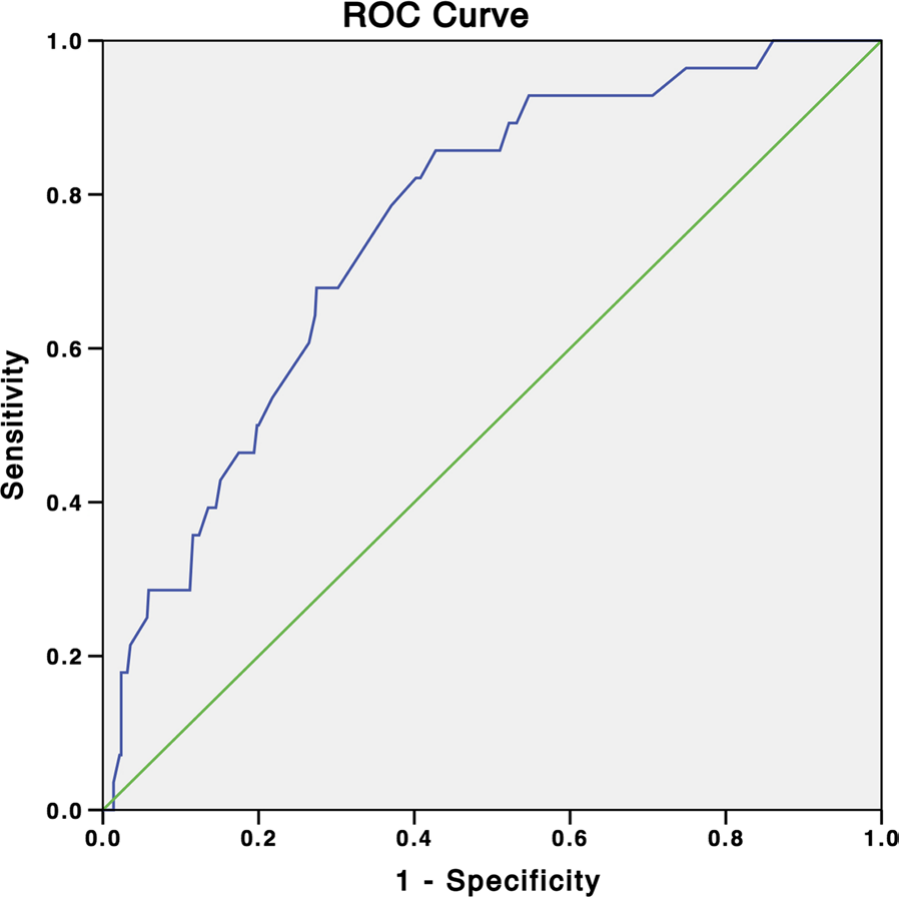
The ROC curve analysis of Hb’s prediction of ISR. ISR, in-stent restenosis; Hb, hemoglobin; ROC, receiver operating characteristic

Predictors of ISR at follow-up were assessed using binary logistic regression analysis. Potentially correlating and clinically important variables were included in the univariate logistic model (Table 2). Diabetes mellitus (OR, 2.588; 95% CI, 1.176–5.696; *P* = 0.018), anemia (OR, 4.283; 95% CI, 1.949–9.410; *P* < 0.001), CKD (OR, 3.058; 95% CI, 1.289–7.252; *P* = 0.011), multiple stenting (OR, 2.592; 95% CI, 1.205–5.573; *P* = 0.015), bifurcation lesion (OR, 2.669; 95% CI, 1.236–5.763; *P* = 0.012), and calcification (OR, 3.529; 95% CI, 1.131–11.014; *P* = 0.030) were predictors of ISR. LDL-c (OR, 1.651; 95% CI, 1.187–2.296; *P* = 0.003) and stent diameter (OR, 3.413; 95% CI, 1.361–8.547; *P* = 0.009) were also associated with ISR, and propensity score matching (Table 3) identified anemia as an independent risk factor for ISR (*P* = 0.009). ROC curve analysis of the diagnostic accuracy of Hb for ISR produced an area under the curve (AUC) of 0.758 (95% CI, 0.675–0.840; *P* < 0.001) (Fig. 1).

**Table 2:** Univariable logistic regression analysis for the prediction of ISR

Variable	OR	95% CI	*P* value
Gender	0.708	0.319–1.571	0.396
Age	1.003	0.967–1.042	0.858
Current smoker	1.020	0.473–2.199	0.961
BMI	0.908	0.788–1.046	0.179
Hypertension	1.186	0.544–2.587	0.668
Diabetes mellitus	2.588	1.176–5.696	0.018
LDL-c	1.651	1.187–2.296	0.003
TG	0.813	0.489–1.353	0.426
LVEF	0.958	0.916–1.001	0.057
Anemia	4.283	1.949–9.410	< 0.001
CKD	3.058	1.289–7.252	0.011
ACS	0.948	0.442–2.033	0.890
Multiple stenting	2.592	1.205–5.573	0.015
Stent length (mm)	0.974	0.908–1.044	0.456
Stent diameter (mm)	3.413	1.361–8.547	0.009
Multivessel coronary disease	1.176	0.464–2.980	0.732
LMCA	3.846	0.801–18.460	0.092
LAD	1.933	0.794–4.707	0.146
LCX	2.191	0.992–4.840	0.052
RCA	0.508	0.212–1.218	0.129
Bifurcation lesion	2.669	1.236–5.763	0.012
Calcification	3.529	1.131–11.014	0.030
Statins	0.727	0.164–3.223	0.674
Aspirin	NA	NA	NA
Clopidogrel	0.853	0.397–1.828	0.683
Ticagrelor	0.693	0.314–1.530	0.364

*ISR* In-stent restenosis; *BMI* body mass index; *CKD* chronic kidney disease; *LVEF* left ventricular ejection fraction; *ACS* acute coronary syndrome; *LDL-c* low-density lipoprotein cholesterol; *TG* triglycerides; *LMCA* left main conronary artery; *LAD* left anterior descending artery; *LCX* left circumflex artey; *RCA* right coronary artery

**Table 3:** Anemia as an independent predictor of ISR after matching on the propensity score

Variable	Before matching		*P* value	After Matching		*P* value
	Anemia n = 179	No anemia n = 359		Anemia n = 159	No anemia n = 159	
Male, n (%)	147 (82.1)	237 (66.0)	0.000	132 (83.0)	132 (83.0)	1.000
Age (years)	62.9 ± 11.2	61.8 ± 9.7	0.139	62.8 ± 11.2	62.3 ± 9.2	0.472
Current smoker, n (%)	89 (49.7)	144 (40.1)	0.034	77 (48.4)	82 (51.6)	0.575
BMI (kg/m^2^)	24.5 ± 2.9	24.4 ± 2.8	0.887	24.7 ± 2.9	24.5 ± 2.8	0.483
Hypertension, n (%)	117 (65.4)	230 (64.1)	0.767	103 (64.8)	103 (64.8)	1.000
Diabetes mellitus, n (%)	42 (23.5)	71 (19.8)	0.323	35 (22.0)	35 (22.0)	1.000
LDL-c (mmol/L)	2.5 ± 1.0	2.6 ± 0.9	0.175	2.6 ± 1.0	2.5 ± 0.9	0.629
TG (mmol/L)	1.5 ± 1.0	1.6 ± 0.9	0.413	1.5 ± 0.8	1.5 ± 0.9	0.805
Hb (g/dL)	110.8 ± 14.8	145.5 ± 13.8	0.000	111.2 ± 14.4	139.5 ± 9.9	0.000
LVEF (%)	59.8 ± 6.0	59.9 ± 7.0	0.859	60.1 ± 6.0	60.6 ± 7.4	0.545
CKD (%)	31 (17.3)	36 (10.0)	0.016	21 (13.2)	14 (8.8)	0.210
ACS (%)	96 (53.6)	199 (55.4)	0.693	89 (56.0)	80 (50.3)	0.312
Multiple stenting, n (%)	52 (29.1)	104 (29.0)	0.984	46 (28.9)	49 (30.8)	0.713
Stent length (mm)	29.6 ± 5.2	29.5 ± 5.6	0.834	29.8 ± 5.3	30.0 ± 5.5	0.640
Stent diameter (mm)	3.1 ± 0.5	3.2 ± 0.5	0.130	3.1 ± 0.5	3.2 ± 0.5	0.762
Multivessel coronary disease (%)	27 (15.1)	75 (20.9)	0.105	26 (16.4)	28 (17.6)	0.765
LMCA, n (%)	3 (1.7)	9 (2.5)	0.759	3 (1.9)	3 (1.9)	1.000
LAD, n (%)	17 (9.5)	65 (18.1)	0.009	16 (10.1)	20 (12.6)	0.479
LCX, n (%)	86 (48.0)	162 (45.1)	0.522	79 (49.7)	77 (48.4)	0.822
RCA, n (%)	73 (40.8)	136 (37.9)	0.516	60 (37.7)	60 (37.7)	1.000
Bifurcation lesion, n (%)	40 (22.3)	98 (27.3)	0.215	39 (24.5)	40 (25.2)	0.897
Calcification, n (%)	12 (6.7)	15 (4.2)	0.206	9 (5.7)	11 (6.9)	0.644
Statins, n (%)	171 (95.5)	338 (94.2)	0.504	151 (95.0)	151 (95.0)	1.000
Aspirin, n (%)	179 (100)	359 (100)	0.019	159 (100)	157 (98.7)	0.156
Clopidogrel, n (%)	93 (52.0)	175 (48.7)	0.483	79 (49.7)	87 (54.7)	0.369
Ticagrelor, n (%)	73(40.8)	164(45.7)	0.281	69(43.4)	65(40.9)	0.650
ISR	19 (10.6)	9 (2.5)	0.000	16 (10.1)	4 (2.5)	0.009

*ISR* In-stent restenosis; *BMI* body mass index; *CKD* chronic kidney disease; *Hb* hemoglobin; *LVEF* left ventricular ejection fraction; *ACS* acute coronary syndrome; *LDL-c* low-density lipoprotein cholesterol; *TG* triglycerides; *LMCA* left main conronary artery; *LAD* left anterior descending artery; *LCX* left circumflex artey; *RCA* right coronary artery

## Discussion

Although the association between preprocedural anemia and poor outcomes post-PCI in patients with CAD is common, data on the impact of anemia on ISR at follow-up is scant. In this study, we established a strong association between anemia and the occurrence of ISR, which could be used as an independent predictor of ISR at follow-up.

Catakoglu et al. [16] demonstrated that anemia is a crucial risk factor for predicting nonfatal coronary events, including ST-segment elevation MI, non-ST-segment elevation MI, target vessel revascularization (TVR), and target lesion revascularization (TLR), post-PCI, whereas Geng et al. [17] established no statistically significant relationship between low hemoglobin levels and ISR. In our research, after adjusting for confounding factors, we substantiated a strong link between anemia and ISR, indicating that anemic patients were more likely to develop ISR after undergoing PCI.

How ISR operates in patients with anemia who undergo PCI is not well-known but probably multifactorial. The presence of anemia could lead to a decreased oxygen supply, and hypoxia has been identified with vascular cell proliferation and angiogenesis [18]: both are necessary conditions for the development, maintenance, and expansion of neointimal lesions in restenosis [19–21]. Compensatory consequences of hypoxia – such as a hyperdynamic state with increased cardiac output, left ventricular hypertrophy, and progressive cardiac enlargement – and a proatherogenic role also contribute to restenosis [22]. Moreover, anemia correlates independently with high platelet reactivity in patients with DES PCI [23], contributing to the development of restenosis [24, 25]. Most recently, a study demonstrated an association between anemia and higher levels of inflammatory markers, such as high sensitive C-reactive protein, fibrinogen, and serum amyloid A, with the authors pointing out that a relationship between anemia and disease outcomes could be caused by underlying inflammation [26]. Because inflammation response is one of the most critical mechanisms for ISR, it is reasonable to infer that anemia could contribute to the progress of ISR. In addition, lower hemoglobin levels are associated with the dysregulation of endothelial cells, causing ISR as well [27, 28].

We also showed in this investigation that higher LDL-c levels, multiple stenting, bifurcation lesion, and smaller stent diameter were linked to post-PCI ISR, which is consistent with previous findings [29–31].

Smoking is a risk factor for CAD, but we found no noteworthy relationship between smoking and ISR. Interestingly, previous studies have reported data supporting the theory that smoking is associated with a lower rate of restenosis, a finding that remains controversial [32]. Diabetes mellitus is considered the most consistent clinical parameter that increases the risk of restenosis [33, 34], which matches our findings.

Multiple studies have also explored the impact of CKD on post-PCI ISR, with the end-stage renal disease found to possibly increase the risk of restenosis occurrence [35], and mild or moderate CKD having no bearing on the incidence of ISR post-PCI [36], which is inconsistent with our results too, one in which patients with CKD were more likely to have anemia.

The current guidelines stipulate a 6 to 12-month DAPT with a class I recommendation after PCI [37, 38]. The duration of DAPT depends on the balance between ischemia and bleeding risk. However, both prolonged and shortened usage of DAPT in patients with CAD undergoing PCI notably affect cardiovascular events. A multicenter prospective real-world study revealed that prolonged DAPT with low-dose ticagrelor was effective and safe, with low incidences of MACE, MI, and stroke/transient ischemic attack, and no major bleeding [39]. P2Y_12_ inhibitor monotherapy after coronary revascularisation has also been assessed in several trials, with one meta-analysis recently establishing that P2Y_12_ inhibitor monotherapy was associated with a similar risk of death, MI, or stroke and lower risk of major bleeding as DAPT, suggesting that shortening DAPT to 1 to 3 months post-PCI could be effective and safe [40]. In this study, we found no difference in the impact of DAPT on patients in the ISR and non-ISR groups, with patients with anemia more likely to stop using aspirin, partly because they were concerned about bleeding.

For restenosis within DES, drug-coated balloon (DCB) and DES are both approved with a class I recommendation [38]. Bioresorbable vascular scaffolds (BVS) have also been proposed as a treatment for ISR, with one study showing that BVS is effective in the treatment of ISR and has an acceptable target lesion failure rate [41]. However, the 1-year follow-up results of a BIORESOLVE-ISR study revealed that BVS increases the rate of device-oriented cardiovascular events compared to DES but has a similar rate to DCB [42]. In our study, 20 patients underwent repeat revascularization: 19 were treated with DCB and one with DES. But our findings and those reported are not sufficient for a generalized outcome. Therefore more studies must be conducted to determine the long-term outcomes of different procedures on ISR treatment.

Radial artery is the recommended default vascular access for PCI [38, 43]; however, crossover from radial to femoral access is sometimes required. One recently published investigation showed that crossover from radial to femoral access diminished the bleeding benefit offered by the radial access site but did not increase the risk of MACE incidence [44]. The authors also developed a simple risk score – MATRIX score – to predict radial crossover in patients with ACS, which could improve outcomes [45]. For patients with ISR, the initial access site selection remains essential to improving management and outcomes. In our cohort, all procedures completed were transradial; the long-term prognosis needs, therefore, to be investigated further.

Despite its promises, our investigation has several limitations. First, it was retrospective in nature. Second, the sample size was relatively small, the follow-up period was fairly short, and the patients involved were from a single hospital; the results, therefore, do not represent a general scenario. Third, the loss of follow-up and the exclusion of patients who refused to be enrolled in the study might have led to a bias. Fourth, our findings were based on a single measurement of preprocedural Hb levels, with Hb during the follow-up not evaluated. Finally, some covariates, such as smoking and LDL-c, might have changed during the follow-up period, in turn affecting our inferences. As a result, larger prospective trials must be conducted to establish anemia’s potential as an independent predictor of ISR.

## Conclusion

Patients with baseline anemia before PCI have a higher incidence of ISR after PCI than their nonanemic counterparts. Anemia could, therefore, be used as a predictor of ISR. Our results have important clinical implications, with the assessment of Hb levels also crucial during periprocedures.

## Data Availability

The datasets generated and analyzed during the present study are not publicly available because of the restrictions by the First Hospital of Jiaxing, but are available from the corresponding author on reasonable request.

## Abbreviations

Hb: Hemoglobin
BMI: Body mass index
CKD: Chronic kidney disease
LVEF: Left ventricular ejection fraction
ACS: Acute coronary syndrome
LDL-c: Low-density lipoprotein cholesterol
TG: Triglycerides
ISR: In-stent restenosis
